# Basic Emotions in Human Neuroscience: Neuroimaging and Beyond

**DOI:** 10.3389/fpsyg.2017.01432

**Published:** 2017-08-24

**Authors:** Alessia Celeghin, Matteo Diano, Arianna Bagnis, Marco Viola, Marco Tamietto

**Affiliations:** ^1^Cognitive and Affective Neuroscience Laboratory, Department of Medical and Clinical Psychology, Center of Research on Psychology in Somatic Diseases, Tilburg University Tilburg, Netherlands; ^2^Department of Psychology, University of Turin Turin, Italy; ^3^Centre for Neurocognition, Epistemology and Theoretical Syntax, Scuola di Studi Superiori Pavia Pavia, Italy; ^4^Faculty of Philosophy, Vita-Salute San Raffaele University Milan, Italy; ^5^Department of Experimental Psychology, University of Oxford Oxford, United Kingdom

**Keywords:** basic emotions, fMRI meta-analysis, lesion studies, blindsight, visual awareness, pluripotentiality, neuropsychology

## Abstract

The existence of so-called ‘basic emotions’ and their defining attributes represents a long lasting and yet unsettled issue in psychology. Recently, neuroimaging evidence, especially related to the advent of neuroimaging meta-analytic methods, has revitalized this debate in the endeavor of systems and human neuroscience. The core theme focuses on the existence of unique neural bases that are specific and characteristic for each instance of basic emotion. Here we review this evidence, outlining contradictory findings, strengths and limits of different approaches. Constructionism dismisses the existence of dedicated neural structures for basic emotions, considering that the assumption of a *one-to-one* relationship between neural structures and their functions is central to basic emotion theories. While these critiques are useful to pinpoint current limitations of basic emotions theories, we argue that they do not always appear equally generative in fostering new testable accounts on how the brain relates to affective functions. We then consider evidence beyond PET and fMRI, including results concerning the relation between basic emotions and awareness and data from neuropsychology on patients with focal brain damage. Evidence from lesion studies are indeed particularly informative, as they are able to bring correlational evidence typical of neuroimaging studies to causation, thereby characterizing which brain structures are necessary for, rather than simply related to, basic emotion processing. These other studies shed light on attributes often ascribed to basic emotions, such as automaticity of perception, quick onset, and brief duration. Overall, we consider that evidence in favor of the neurobiological underpinnings of basic emotions outweighs dismissive approaches. In fact, the concept of basic emotions can still be fruitful, if updated to current neurobiological knowledge that overcomes traditional *one-to-one* localization of functions in the brain. In particular, we propose that the structure-function relationship between brain and emotions is better described in terms of pluripotentiality, which refers to the fact that one neural structure can fulfill multiple functions, depending on the functional network and pattern of co-activations displayed at any given moment.

## Introduction

Any textbook on neuroscience, psychobiology or neuropsychology includes a chapter with a summary on emotions. Although extensively studied, an unequivocal definition of emotions is still lacking and the subject of contentions. For example, in the 1980’s, Fehr and Russell (1984, p. 464) wrote that “Everyone knows what an emotion is, until asked to give a definition. Then, it seems, no one knows.” Kleinginna and Kleinginna (1981) considered 92 definitions and 9 skeptical descriptions produced by scientists in the field, which effectively represent the lack of consensus with regard to the characteristics that define the concept of emotion and its usefulness in the scientific framework.

Clearly, differences and idiosyncrasies in relation to the general concept of emotions are reflected in the construct of ‘basic emotions’; a view that purports the existence of a small number of so-called primary emotions, usually comprising fear, anger, joy, sadness, surprise and disgust. Indeed, there are different theories of, and different approaches to, basic emotions, as well as variable taxonomies of emotions, which are not entirely superimposable (Galati, 1993; Tracy and Randles, 2011). Besides specific differences, supporters of the existence of basic emotions, such as Ekman, Tomkins, Izard, Plutchik, Levenson, and Panksepp share some fundamental assumptions derived from an evolutionistic Darwinian approach (Tomkins, 1962; Plutchik, 1980; Izard, 1994; Levenson, 1994; Panksepp, 1998; Ekman, 1999). This approach suggests that emotions have developed and got selected because of their adaptive value, meaning that, through some automatic mechanisms, they are capable of regulating the interaction with the proximal environment, while at the same time providing effective responses, both instrumental and communicative, in relation to the relevant situation for survival (Tooby and Cosmides, 1990; Shariff and Tracy, 2011).

In contrast, psychological construction theories argue against the innateness of emotions. These theories emphasize that the different types of emotions emerge from a construction process. That is, basic psychological operations, such as perception, attention and memory, combine to generate an emotional meaning that is influenced by social and linguistic factors (Barrett and Russell, 2015). A particular psychological construction view, the Conceptual Act Theory (CAT) (Barrett et al., 2015; Barrett, 2017), claims that each emotional episode is built up by the brain from the combination of core affect (a representation of raw sensations related to the body) and a categorization process based upon prior experience and mediated by conceptual and linguistic knowledge (Barrett, 2006, 2014, 2017).

This longstanding debate has been revitalized by a series of quantitative meta-analyses drawing on the impressive amount of data produced by functional magnetic resonance (fMRI) and positron emission tomography (PET) studies. The goal of these meta-analyses was to examine the invariance of the relationship between certain neural structures and some basic emotions. Since it is on this methodological ground that the constructionist approach has recently concentrated to draw arguments on the non-existence of the neural correlates of basic emotions, we will start with a brief review of these fMRI/PET meta-analytical studies. Then, we will consider which model of relationship between neural structures and psychological functions supports the criticisms of the constructionist approach to basic emotions. We will propose an approach that retains the value of basic emotions, but revises some drawbacks and confers better neurobiological plausibility to this concept. Lastly, we will review other neuroscientific evidence that, in our opinion, is consistent with the existence of basic emotions.

## Terminological Considerations

Emotions are often conceived as composite and multi-component constructs, including the evaluation of an external stimulus, neural responses and the related psychophysiological reactions, expression modifications, instrumental actions and, lastly, experiential and subjective psychological components related to such changes (see Scherer, 2009; Adolphs, 2017 for examples). As a function of the multi-componential nature of the more general concept of emotion, the basic-ness of emotions could be investigated following three different profiles: conceptual, psychological, and biological (Ortony and Turner, 1990; Scarantino and Griffiths, 2011).

From a conceptual perspective, the notion of basic emotions refers to logical-formal criteria that define the existence of some categories within taxonomies. In that regard, a concept is considered to be basic if it contributes to create the most abstract category within a hierarchy where the elements share a certain number of common properties that are sufficient to determine whether a single element belongs to that category (Scarantino and Griffiths, 2011). For example, following the works of Rosch (1973), the category “dog” is a basic category because it represents the most abstract category of which it is possible to create a mental image, it is always used by adults, and learned rapidly by children during language learning. The subordinate categories (i.e., German Shepherd, Dachshund) share some common attributes, but these are not very differentiated. The superordinate categories (i.e., mammals) share few common attributes and differ greatly from one another.

From a psychological perspective, an emotion is basic only if it does not contain another emotion; that is, if it represents an atomic, irreducible psychological construct. Emotions that are not deemed basic are variously interpreted as resulting from the integration of basic emotions, or from the integration of basic emotions and cognitive functions (but see Pessoa, 2010 on the fuzzy distinction between emotion and cognition; and LeDoux and Brown, 2017 for emotions as higher-order states integrated with cognition). For example, hostility can be considered a mix of anger and disgust, sociability derives from the combination of joy and acceptance, and guilt melts feelings of pleasure and fear (Plutchik, 1994).

Lastly, by claiming that an emotion is biologically basic, it is assumed that there is an innate, hardwired mechanism that links, for example, the processing of a sensory input that signals potential danger with the production of a coordinated pattern of behavioral responses such as freezing or flight. Arguably, this is the notion of basicness that most researchers have in mind when referring to basic emotions. Ekman (1999) proposed that basic emotions have the following characteristics: (1) distinctive universal signals (e.g., facial expressions); (2) universal and distinct antecedents (e.g., the sight of a snake in the grass); (3) characteristic physiological correlates; (4) are induced by an automatic processing (i.e., non-conscious or involuntary); (5) emerge early in ontogeny; (6) are present in other non-human primates; (7) have rapid onset; (8) are of short duration; (9) are not controlled voluntarily; (10) are associated with distinctive thoughts, memories and images, as well as with (11) distinctive subjective experience.

Although it may appear that the conceptual, psychological, and biological approaches to basic emotions converge, this is not necessarily the case. Moreover, different meanings assigned to the same word, for example ‘fear,’ can cause additional confusion. In this respect, LeDoux (2012, 2014; LeDoux and Brown, 2017) has clarified the confusion that can arise when we conflate terms that refer to different processes, i.e., processes related to the conscious experience with those that refer to the reflex-like processing of stimuli and the triggering of responses, and when assuming that the brain mechanism that underlie the two types of processes are the same. From a neuroscientific perspective, the biological basicness of emotions holds meaning and value only if neurobiological underpinnings characteristically associated to different instances of emotions can be found. Indeed, while Ekman (1999, p. 50) never addressed the issue directly, he nevertheless posited that “there must be *unique* physiological patterns for each emotion, and these (central nervous system) patterns should be specific to these emotions not found in other mental activity” (emphasis in the original).

## Meta-Analytic Studies

Meta-analysis is used to quantitatively assess the results of a set of studies and evaluate the replicability and statistical robustness of individual data studies, which are often based on limited sample size and statistical power (Wager et al., 2009; Radua et al., 2012). Meta-analysis thus adopts a specific method to address the common theme of cognitive and affective neuroscience; that is, to define the relationship between neural structures and mental functions; emotions the present case. The first meta-analysis on the neural correlates of emotions was conducted on 55 neuroimaging studies (PET and fMRI) to determine whether the different emotions present common or specific neural activation patterns and, in the second case, which brain regions are associated with each emotion (Phan et al., 2002). The semi-quantitative analysis enabled isolating different brain regions associated with different emotions. In particular, the processing of fear and sadness was associated with activation of the amygdala and cingulate cortex, respectively, while joy and disgust were associated with increased metabolic activity in the basal ganglia (Phan et al., 2002). Conversely, the mesial prefrontal cortex was generally and extensively involved in all the emotions studied. These results were partly confirmed by a subsequent meta-analysis of 106 PET and fMRI studies (Murphy et al., 2003). The authors found that specific brain regions were activated for fear (amygdala), disgust (insula and globus pallidus) and anger (lateral orbitofrontal cortex) and reported activations in the cingulate cortex for fear and, in part, for joy. The findings of these first meta-analyses thus appear to be fairly coherent with physiology and neuropsychological data from animals, which have revealed deficits in the recognition and expression of specific basic emotions as a result of focal lesions in the areas reported by the meta-analyses (see below).

Over the years, more sophisticated methods have been implemented that have helped to make the results of meta-analyses more precise from an anatomical as well as statistical point of view. In particular, these new methods enable to maintain the spatial information related to the original coordinates of activation, instead of converting them into macro-regions to divide the brain into sections of equal volume, as in the earlier works just mentioned. Vytal and Hamann (2010) used the activation likelihood estimation (ALE) technique (Turkeltaub et al., 2002). With this method, it is possible to preserve the three-dimensional spatial coordinates that define the areas activated in the original studies at the level of each voxel, the smallest spatial unit in fMRI. The authors pair-wise compared the activation maps of single emotions (e.g., Fear vs. Anger, Fear vs. Joy, etc.), showing that each basic emotion is associated with a distinct pattern of brain activity. More precisely, the results indicate that fear is related to activation of the amygdala and insula, anger to orbitofrontal cortex, disgust to anterior insula, the ventral prefrontal cortex and the amygdala, happiness to activation of the rostral anterior cingulate cortex and sadness to the medial prefrontal cortex and the caudal anterior cingulate cortex. Note, therefore, that the study by Vytal and Hamann (2010) did not report a one-to-one relationship between structure and function, nor did it uniquely associate an emotion with a single neural structure, but with a network that may contain structures involved in the processing of more than one basic emotion. Kirby and Robinson (2015) replicated Vytal and Hamann results regarding consistency and specificity across basic emotions, using the BrainMap database to undertaking an ALE meta-analysis on each emotion (Laird et al., 2005). They concluded that a neural profile for each basic emotion seems to exist, and they suggested that a multi-system model with distributed networks differentiating each emotion should replace the traditional locationist approach.

The results of the meta-analysis conducted by the group led by Lisa Feldman Barrett and Tor Wager, instead, have been interpreted as supporting a constructionist view of emotions (Kober et al., 2008; Lindquist et al., 2012; Wager et al., 2015). Kober et al. (2008) performed a data-driven co-activation analysis on every study on emotion, including those that did not distinguish between different emotion categories, and found six functional groups. These clusters are considered as the original and primitive neurofunctional components of “basic psychological operations” (Lindquist et al., 2012) from whose interaction and integration emotions emerge. They include circuitry associated with cognitive and motor functions, such as language and executive functions, conceptualization, or visual functions. Lindquist et al. (2012) considered only studies on discrete emotion categories, and analyzed the density of activation in areas of 10 mm which significantly respond to an emotion in comparison with the activity elicited through the mean of all other emotions in the same area [e.g., Fear vs. (Anger + Happiness + Sadness + Disgust)/4]. These authors reported that it was not possible to isolate unique and specific neural correlate for each basic emotion, because each area activated by one emotion was also activated by at least another basic emotion. Recently, Wager et al. (2015) have proposed a multivariate meta-analysis based on a hierarchical Bayesian approach. In addition to generating summary brain activation maps for each emotion, as was already the case in previous studies, this method was also able to predict the number and position of activations from a single study and to calculate the probability that a new study will contain peaks of activations within a particular brain region. Although analyses have revealed that “each emotional category is associated with a unique and prototypical pattern of activity distributed across multiple regions” (p. 1), the authors interpret the data as disconfirming basic emotions theories, as these activation patterns partly overlap one another, and are linked to other functions considered *primitive*, as already reported in previous work by the same group.

Clearly, the choice of statistical methods in meta-analysis is crucial and can lead to different results because diverse analyses inevitably bring assumptions that may emphasize some trends in the data and underestimate others, even though raw data are not very different. For example, Vytal and Hamann (2010) used a standard pairwise comparison, Lindquist et al. (2012) conducted a density analysis, and Kober et al. (2008) a co-activation approach. Besides methodological differences, it is important to bring into focus the criterion implicitly adopted from the outset to accept or reject the concept of basic emotions – the existence of a univocal relationship between the neural structure and emotions – and, consequently, the interpretation of the results, as we will outline in the next session.

## Limitations of Neuroimaging Studies

What inferences can be made on the basis of fMRI studies? What are the limitations of the methodology with respect to the debate on the existence of basic emotions?

A first limit is epistemological. That is, imaging studies are good at revealing which neural structures are *involved* in the processing of basic emotions, but are silent with respect to what structures are *necessary* to recognize or express such emotions. In this sense, they offer a type of ‘weak’ or correlational evidence and should be interpreted in the light of other data, such as lesion studies, in which the correlational nature of fMRI data is elevated to a causal inference (Krakauer et al., 2017). Others and we tend to believe that the starting point to understand the neurobiology of emotions is the analysis of behavior, as we cannot rely solely on the correlational approach of neuroimaging data devoid of relation with behavioral outcomes. Indeed, the causal-mechanistic explanations are qualitatively different from understanding how component modules perform the computations that then combine to produce behavior (Krakauer et al., 2017). In order to investigate and understand emotions we need categories as well as we need to make distinctions among brain processes, albeit current categories represented by English words such as “fear,” “anger,” or “disgust” may be too simplistic (Adolphs, 2017).

To sum up, neuroimaging data and recent meta-analyses do not seem to us to provide sufficiently solid ground for rejecting the existence basic emotion at the neurobiological level. In addition, other features considered typical of basic emotions, like automaticity or early onset during sensory processing (Ekman, 1999; see above), are not considered in these studies. Second, meta-analytic methods combine studies conducted under different experimental conditions to highlight ‘neural regularities.’ To this end, they dampen methodological differences of studies that have investigated certain basic emotions and that, owing to such methodological specificities, may have given rise to idiosyncratic activations. Most of the studies on the neural correlates of basic emotions actually assessed the activations in response to visual recognition of certain emotional stimuli, typically facial expressions. Other studies used different visual or auditory stimuli, or asked subjects to imagine emotional situations and so on, thereby investigating different aspects and functions of emotional phenomena. The choice to tackle the neural bases of basic emotions by pooling together studies so different involves strong and often implicit assumptions with respect to the nature of emotions. It is plausible that an a-modal core exists, which responds to emotional content regardless of the type of stimulus or the sensory modality that elicits such emotions, or irrespectively of whether we are evaluating a mental image or an experience induced by the evaluation of a complex situation, perhaps presented verbally, with cartoons, and so on (Schirmer and Adolphs, 2017). This core was indeed reported in the meta-analysis by Feldman Barrett and Wager, and attributed to the paralimbic and limbic areas (Kober et al., 2008; Lindquist et al., 2012). But it is not obvious that such a-modal core can also represent the ‘neural marker’ that distinguishes several basic emotions from others, if the specific characteristics related to stimulus events and other properties of the phenomenon are averaged from the outset. These specificities should not, in fact, necessarily be considered ‘noise,’ and it is worth remembering that one of the characteristics considered typical of basic emotions is the presence of specific behavioral responses and antecedents. In other words, it seems unlikely that there is a fearful response regardless of specific sensory events that cause it, the expressive and instrumental responses associated with it and so on, such as meta-analytic approaches are led to implicitly assume. Thus, differences in such events may explain, at least partly, the variability in the activation patterns related to specific basic emotion categories.

## The Structure-Function Relation and Basic Emotions

**FIGURE 1:**
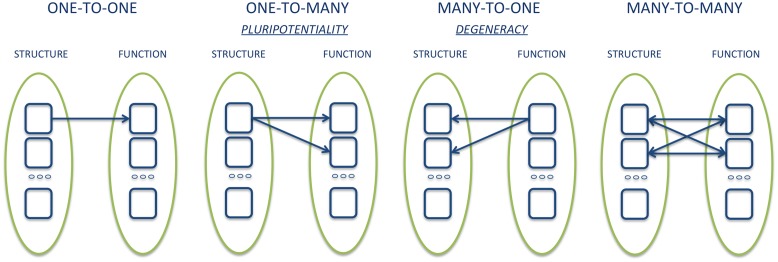
Graphical example of different types of structure-function relationships.

As noted above, the results of some meta-analyses, such as those by Vytal and Hamann (2010) and Lindquist et al. (2012), while reporting similar results, came to very different conclusions. What, then, counts as evidence for deciding whether the results of neuroimaging support the concept of basic emotions? One important argument often cited for denying the existence of basic emotions at the neurobiological level is that meta-analysis data have shown that each area activated by a basic emotion is also activated by at least another emotion. Since it is not possible to identify brain regions that are consistently and uniquely associated with only one emotion, the latter does not have dedicated neural underpinning. Such reasoning assumes that basic emotion theorists are committed to one-to-one relationship between individual brain areas and mental functions. However, it is becoming increasingly clear that the correspondence between brain structure and function is better represented by a one-to-many relationship, or pluripotentiality (Gallese and Lakoff, 2005; Dehaene and Cohen, 2007; Gallese, 2008; Hurley, 2008; Pessoa, 2008, 2014; Anderson, 2016). For example, Anderson et al. (2013) have shown that even the smallest region of the brain is involved in a multiplicity of cognitive functions and behavioral categories. Accordingly, the elementary unit of analysis seems to move from the single neural structure to the distributed network of dynamic interactions between structures (Bressler and Menon, 2010). The functional role of a single structure is then determined in part by the interactions that such a structure entertains with other regions at a given time (Klein, 2012; Scarantino, 2012; Pessoa, 2017). The network thus assumes a modality defined as dominant, or a function tends to be expressed with greater probability through the interaction of certain brain regions, which may, however, contribute to the expression of a different task or function when interacting with other structures (Pessoa, 2014) (**Figure 1**).

It is interesting to note that if the strict criterion of a one-to-one relationship between structure and function adopted to criticize basic emotions were adopted in other domains, very little would remain of the knowledge that underpins cognitive neurosciences. For example, it is known that auditory stimuli also activate the primary visual cortex (V1) (Pockett et al., 2013), and V1 also responds to tactile stimuli (Nordmark et al., 2012). Likewise, the primary motor area determines not only voluntary movements, but is also active in various tasks that do not require the execution or planning of any movement, such as working memory, language, visual and auditory tasks (Kukleta et al., 2016; Tomasino and Gremisse, 2016). Moreover, Broca’s area, typically associated with speech production, is also involved in various tasks such as preparing the movement and action (Anderson, 2010). If we were to take this logic to the extreme consequences, we would come to the paradoxical conclusion of having to abandon concepts like vision, speech or hearing since there is not a strict one-to-one relationship between these functions and the neural structures associated with them.

Does the basic emotion view entail a one-to-one mapping between single brain regions and each basic emotion? Indeed, not even Ekman (1999) assumed that each basic emotion is associated with a single brain area; rather, he explicitly spoke of *patterns* of activity, which is in fact what some neuroimaging meta-analyses found. The constructionists, however, deny that such patterns constitute legitimate neural bases for basic emotions, as they do not correspond to intrinsic patterns of neural co-activation, i.e., to the networks found during resting state (Touroutoglou et al., 2015). Constructionists suggest that only these networks may reveal the “basic psychological operations” (Lindquist et al., 2012).

Undoubtedly, the concept of basic emotions requires updating and reformulation according to more recent neurobiological principles about brain functioning, including those outlined above. However, abandoning the one-to-one mapping between structures and functions does not lead, in principle, to abandon basic emotions themselves. In fact, “basic psychological operations,” which have been proposed as the primitive and elemental constituents of emotions and other mental states (Lindquist et al., 2012), also seem to necessitate a clearer operationalization. These functions are supposed to be primitive insofar as they are linked to ‘intrinsic’ brain networks at rest, and hence do not hinge on overt behavior. Beyond the limbic and paralimbic structures that represent the emotionally undifferentiated core, the authors grouped the remaining brain structures into four clusters: (1) cognitive and motor clusters related to language functions, executive and attentional; (2) the posterior mesial regions cluster related to conceptualization; (3) the mesial prefrontal cluster, also linked to conceptualization; (4) the visual occipital cluster. It is not obvious that these categories have unique neural correlates, and can guide research on the relationship between neuronal structures and mental functions, as well as the comparison between animal and human neuroscience, better than basic emotions. Which definition is necessary and sufficient to make “conceptualization” neurobiologically founded? How many tasks are the mesial prefrontal structures involved in, or how many non-linguistic tasks are the structures of the inferior frontal gyrus, typically involved in speech production, involved in?

An example of how the shift of focus from the individual structures to networks can be heuristically useful in the study of the neural correlates of basic emotions is offered by recent fMRI studies using multivariate techniques (MVPA) or investigating the dynamic relationships between networks, as a function of different emotions. Saarimäki et al. (2016) classified the activities of each voxel in response to six basic emotions, showing that they are associated with distinct neural networks, though widely distributed in the brain (but see Clark-Polner et al., 2017 for a different interpretation). Another recent study has investigated whether and how the amygdala functional connections vary depending on the presentation of different facial expressions of basic emotions (Diano et al., 2017b). The amygdala is in fact traditionally considered to be involved in the processing of fearful signals. However, several neuroimaging studies and some meta-analyses have reported the involvement of the amygdala in the perception of other basic emotions such as anger, sadness or joy (Sergerie et al., 2008; Kirby and Robinson, 2015). This result was initially interpreted as disconfirming the functional specificity of the amygdala and in contradiction to basic emotions views. However, applying a method to study the dynamic changes of the functional connections that the amygdala entertains with the rest of the brain, we observed that the amygdala recruits different structures in response to the various basic emotions, so as to constitute a characteristic functional network for each emotion (Diano et al., 2017b). For example, during fear processing, amygdala activity was specifically correlated with activity in posterior visual areas including V1, fusiform gyrus and superior temporal sulcus, whereas processing of happiness involved co-activations with more anterior regions, such as dorso-medial prefrontal cortex (dmPFC) and anterior cingulate cortex (ACC).

The pluripotentiality of the structure-function relationship and the adoption of network level of analysis as significant units, instead of individual regions, also raise the need to investigate the temporal dimension more closely. In fact, the neural regions that represent the substrate of different basic emotions may, in principle, remain the same, whereas it is the uniqueness of the temporal properties of their connections and the synchrony between them that differentiate between the emotions. The time dimension is indeed a further definitional criterion of basic emotions, as originally conceived. For example, fear, anger or disgust are considered to trigger automatic reflex-like responses to potentially dangerous events for survival, and these responses can be adaptive only to the extent that they can be implemented quickly. Conversely, the speed of responses related to joy or sadness is probably less relevant and these latter emotions can unfold at longer time scales. fMRI has a low temporal resolution (in the order of several seconds) considering the few milliseconds that characterize neural responses. There is therefore the risk of observing only the final responses, and of losing information about the earliest responses. Electroencephalography (EEG) and magnetoencephalography (MEG) are valid methodological alternatives, offsetting a relatively limited spatial resolution compared to fMRI with a time resolution of a few milliseconds. An exhaustive assessment of these techniques is beyond the scope of this paper, but we note that using these techniques it has been possible to investigate and discriminate the temporal and spatial profiles of neural networks activated in response to different basic emotions (Eger et al., 2003; Esslen et al., 2004; Morel et al., 2009; Calvo and Beltrán, 2013; Luo et al., 2013; Costa et al., 2014; Nakamura et al., 2014; Wang and Bastiaansen, 2014; Candra et al., 2015; Liu et al., 2015; Mattavelli et al., 2016; Mavratzakis et al., 2016; Kokinous et al., 2017).

## Basic Emotions and Awareness

Another feature considered characteristic of basic emotions is their automaticity, or the fact that the sensory processing of the triggering events, as well as the expression of the responses associated with them, do not necessarily depend on the awareness (e.g., Ekman, 1999). It is known that only a fraction of sensory input gives rise to conscious perceptions. For example, the stimuli to which we do not pay attention do not become part of our conscious contents (Kentridge et al., 2004). Also, if the energy of the stimulus is too low and below the threshold of sensory detection, or if the stimulus is too short (subliminal), we are not aware of its presence (Savazzi and Marzi, 2002; Dehaene et al., 2006). Emotions, and in particular stimuli that communicate potential threat, however, seem less dependent on attention and awareness. fMRI studies in which attention was manipulated with dual tasks showed that expressions of fear presented outside the attentional focus, and therefore not consciously perceived by the subjects, often activate the amygdala (Vuilleumier et al., 2001; Anderson et al., 2003; Bishop et al., 2004; Williams et al., 2005; but see Pessoa et al., 2002). Other studies used masking or binocular rivalry procedures to block conscious perception of the emotional stimuli (Morris et al., 1998, 1999; Whalen et al., 1998, 2004; Critchley et al., 2002; Killgore and Yurgelun-Todd, 2004; Pasley et al., 2004; Liddell et al., 2005; Williams et al., 2006a,b; Carlson et al., 2009; Yoon et al., 2009; Juruena et al., 2010). Even in this case, expressions of fear that were not consciously perceived activated a subcortical network, typically involving the superior colliculus, the pulvinar and the amygdala (see Tamietto and de Gelder, 2010; Diano et al., 2017a, for reviews). These studies were largely inspired by the seminal work of LeDoux on subcortical pathways to the amygdala involved in the processing of non-conscious fearful stimuli (LeDoux, 1996; LeDoux and Brown, 2017).

Particularly interesting are those studies that instead of experimentally manipulating the attention to or visibility of the stimuli, have studied patients with neuropsychological attentional deficits and/or impaired visual awareness. For example, patients with hemispatial neglect do not pay attention to events in the right side of the space and the stimuli that appear in this side are typically not consciously perceived. However, fear stimuli projected in the right side activate the amygdala and can more easily access conscious awareness than neutral or joyful stimuli (Vuilleumier and Schwartz, 2001; Vuilleumier et al., 2002; Tamietto et al., 2005, 2007, 2015; Domínguez-borràs et al., 2012). Another particularly interesting group of patients are those with ‘blindsight’ (Weiskrantz et al., 1974; Tamietto and Morrone, 2016). Such patients are clinically blind in a portion of the visual field as a result of damage to V1. However, they can discriminate between different expressions, such as joy or fear, show distinctive mimicking responses and specific physiological activation, even though they are not aware of the presence of such stimuli and report subjectively ‘to guess’ (Ladavas et al., 1993; de Gelder et al., 1999, 2008; de Gelder and Tamietto, 2007; Tamietto and de Gelder, 2008, 2010; Tamietto et al., 2009, 2012; Van den Stock et al., 2011b, 2014, 2015; Bertini et al., 2013; Cecere et al., 2013, 2014; Celeghin et al., 2015a,b,c; Georgy et al., 2016). These results challenge a perspective where emotions are generated through linguistic mediation and conceptualization. However, they are not in contrasts with higher-order theories of emotional consciousness that consider the latter as emerging from cortical circuits also involved in cognitive states of consciousness (LeDoux and Brown, 2017). According to this view, the difference between emotional and non-emotional experiences does not parallel the subcortical vs. cortical distinction, as emotional consciousness is not instantiated in subcortical areas involved in the processing of basic sensorial input and responses to affective signals. Rather, subcortical areas provide non-conscious input to cortical networks that implement conscious experiences regardless of their content.

The study of emotional responses in the absence of awareness also offers a privileged perspective from which to evaluate another characteristic feature of basic emotions theories: that of being determined by typical and phylogenetically old stimuli. Until a few years ago, the most commonly used stimuli in affective neuroscience and experimental psychology were images of facial expressions. However, more recent studies have also used body postures (de Gelder, 2006; de Gelder and Hadjikhani, 2006; Tamietto et al., 2009, 2015; de Gelder et al., 2010, 2012; Van den Stock et al., 2011a,b, 2013, 2015). Moreover, stimuli that represent an ancestral danger, such as snakes and spiders (Öhman et al., 2001; Öhman, 2009; Troiani and Schultz, 2013) have also been tested. Typically, all these stimuli can induce specific psychophysiological responses and can activate the amygdala and other structures related to the sensory encoding of potentially dangerous signals (Carlsson et al., 2004; Wendt et al., 2008; Alpers et al., 2009; Almeida et al., 2015; Tamietto et al., 2015). Interestingly, the non-conscious processing of facial expressions related to the so-called social and complex emotions, such as arrogance, guilt or embarrassment, seems to be abolished when visual awareness is lacking, as in patients with blindsight (Celeghin et al., 2016). Similarly, the processing of complex scenario images designed to evoke emotions seems to rely on awareness in order to be able to trigger neural responses and evoke psychophysiological changes typical for that emotion (de Gelder et al., 2002; Grabowska et al., 2011).

In summary, these studies appear to converge in indicating that some biologically primitive stimuli, for which we seem evolutionarily prepared to respond and that are traditionally associated with basic emotions, can be processed in the absence of awareness.

## Basic Emotions and Neuropsychological Patients

The study of patients with focal brain damage provides evidence that complements the findings of neuroimaging studies for several reasons. First, lesion studies offer causal, rather than merely correlational, evidence with respect to the functional role of a given neural structure in mediating behavior, and inform us on how the networks properties are altered by the absence of a particular component. Also, brain damage can alter a function in a completely unexpected and unpredictable way, thus radically changing the way we think about the functional architecture of the mind/brain (Caramazza, 1986). Lastly, the study of the constellation of symptoms resulting from the damage and the possible resolution over time thereof can outline the many-to-one structure-function relationship, owing to plasticity processes and/or neural reorganization (Adolphs, 2016). This aspect, which goes by the name of ‘degeneracy,’ complements the pluripotentiality, and defines the capacity of structurally different elements to implement the same function or generate the same output (Edelman and Gally, 2001; Friston and Price, 2003).

There is a wealth evidence showing that deficits in the recognition of specific emotions result from focal lesions in different brain areas (Calder et al., 2001). Adolphs et al. (1994) first showed how bilateral amygdala damage induces a selective deficit in the recognition of facial expressions of fear. In later studies it was also noted that an impaired ability in these patients to recognize fear is not associated with the normal ability to discriminate other facial features such as identity (Adolphs et al., 1995), gender and age (Anderson and Phelps, 2000). These deficits in the recognition and in the experience of fear as a result of amygdala lesions may extend to other emotions as well as non-facial stimuli, such as vocal expressions (Scott et al., 1997; Calder et al., 2000, 2001; Brierley et al., 2004) body postures (Sprengelmeyer et al., 1999), snakes or spiders (Adolphs et al., 1994, 1995; Bechara et al., 1995; LaBar et al., 1995; Adolphs, 1999; Feinstein et al., 2011). These results are not in contradiction with the neuroimaging data described previously, which also report an involvement of the amygdala for emotions other than fear, as we have already discussed.

Patients with a selective lesion to the anterior insula and basal ganglia, most likely in the pallidum and ventral striatum, show impaired perception of disgust and experience less disgust in response to scenes that describe and reproduce bodily products, violence or repulsive animals (Phillips et al., 1997, 1998; Sprengelmeyer et al., 1998; Calder et al., 2000, 2001; Tremblay et al., 2015). Furthermore, patients with neurodegenerative diseases involving the insula and basal ganglia (such as Huntington’s disease) also show diminished ability to identify the distaste for bad smells (Mitchell et al., 2005) as well as the inability to recognize disgust in other people’s faces (Sprengelmeyer et al., 1996, 1998; Calder et al., 2001; Suzuki et al., 2006; Kipps et al., 2007; Sprengelmeyer, 2007). Similarly, patients receiving anterior insula electrical stimulation report visceral sensations consistent with the experience of disgust (Penfield and Faulk, 1955), and insula stimulation may cause behavioral and physiological responses typical of disgust in both, monkeys (Caruana et al., 2011) and humans (Papagno et al., 2016). Lesion and neuroimaging evidence thus support the idea that basal ganglia take part directly in the processing of disgust, rather than being simply involved by proximity and interconnections with the insula.

Orbitofrontal cortex lesions cause pathological manifestations of anger and lack of self-control, as is well known from the classic case of the patient Phineas Gage (Damasio, 1994). More recent studies have shown that patients with lesions to the orbitofrontal cortex become irritable more easily and use verbal (but not physical) aggression more frequently compared to neurologically healthy subjects (Grafman et al., 1996). Psychopathy and antisocial disorders are marked by an increase in aggression, which relates to a structural (Raine et al., 2000) and functional change in the orbitofrontal cortex (Glenn et al., 2009; Yang et al., 2009; Harenski et al., 2010). One study also found that individuals with borderline personality disorder have lower metabolic activity in the lateral orbitofrontal cortex and are more prone to aggression toward others (Goyer et al., 1994).

All things considered, neuropsychological studies bear inevitable limitations, and cannot be the unique basis to refute or accept any hypothesis about neural architecture of mental functions. The study of selected patients presents with limitations, especially in regard to replicability of the results because of the intrinsic variability (etiology, extension, age) of each patient (e.g., Premi et al., 2016). However, neuropsychology is clearly not committed to build up a different model of antomo-functional correlations for each case studied. What patients studies can reveal is that each patient performance potentially provide a relevant evidence for a model of the neural bases and organization of mental functions (Caramazza, 1986). So far, it appears to us that the bulk of evidence lend support to the existence of relatively segregated neural networks for the processing of different basic emotions.

## Interim Conclusion

In this review, we summarized a number of studies and themes that have recently revitalized the debate about the existence of the neurobiological basis of emotion. We believe that, when considered alone, neuroimaging data are not sufficient to disconfirm the concept of basic emotions, especially when derived from ‘resting state’ experiments. Furthermore, meta-analytical studies appear to be heavily influenced not only by methodological choices, but also by the theoretical assumptions that are not always explicit. The need to previously define which evidence may apply for or against the existence of basic emotions is underlined by the fact that similar results have given rise to conflicting interpretations. The assumption of a one-to-one relationship between neural structures and mental functions is a central argument for the rejection of basic emotions in a neurobiological perspective by those who adopt a constructivist theoretical approach.

We interpret current findings as suggesting that the neurobiological existence of basic emotions is still tenable and heuristically seminal, pending some reformulation. Moving the focus of neuroscientific research from individual brain regions to networks, and from the simplistic region-based one-to-one localizations to more sophisticated network-based one-to-many relationship between neural structure and function seems to prefigure a more modern and neurobiologically plausible approach to the study of basic emotions. Finally, we argued that is important to consider, along with neuroimaging data, evidence from behavior in healthy subjects and patients with focal brain damage. When these findings are considered conjointly, the picture seems to us in favor of the neurobiological existence of basic emotions, albeit in a not entirely univocal way. The discrepancies that still exist reflect methodological limitations, the need to update and reformulate too rigid definitional criteria with respect to what the basic emotions are (Scarantino, 2015), and require a clearer distinction between the psychological, biological and conceptual profiles in ‘basicness’ of emotions.

## Author Contributions

AC, MD, AB, MV, and MT performed bibliography search and discussed results. AC, MD, AB, MV, and MT wrote the paper and approved final version for submission.

## Conflict of Interest Statement

The authors declare that the research was conducted in the absence of any commercial or financial relationships that could be construed as a potential conflict of interest. The reviewer MG and handling Editor declared their shared affiliation, and the handling Editor states that the process nevertheless met the standards of a fair and objective review.
